# Clinical Benefit, Price, and Regulatory Approval of Cancer Drugs Granted Breakthrough Therapy Designation in China, 2020-2024

**DOI:** 10.1001/jamanetworkopen.2024.39080

**Published:** 2024-10-16

**Authors:** Xingxian Luo, Xin Du, Xufeng Lv, Yue Yang, Xiaohong Zhang, Lin Huang

**Affiliations:** 1 Department of Pharmacy, Peking University People’s Hospital, Beijing, China; 2 Vanke School of Public Health, Tsinghua University, Beijing, China; 3 Center for Drug Evaluation, National Medical Products Administration, Beijing, China; 4 School of Pharmaceutical Sciences, Tsinghua University, Beijing, China; 5 Key Laboratory of Innovative Drug Research and Evaluation, National Medical Products Administration, Beijing, China

## Abstract

**Importance:**

The China National Drug Administration (NMPA) established the breakthrough therapy designation (BTD) in 2020 to encourage the accelerated development of drugs for the prevention and treatment of diseases that are life-threatening. However, the differences between BTD and non-BTD cancer drugs regarding clinical benefit, regulatory approval, and price are unclear.

**Objectives:**

To compare BTD and non-BTD cancer drugs in clinical benefit (defined as efficacy and safety), novelty, time to approval, and average monthly treatment price.

**Design, Setting, and Participants:**

This cross-sectional study analyzes the original indication of BTD and non-BTD novel cancer drugs approved by the NMPA between July 8, 2020, and July 8, 2024. Data on efficacy, safety, regulatory approval, and price of cancer drugs were extracted from pivotal clinical trials based on review reports published by the NMPA, peer-reviewed articles or meeting reports, and winning bid prices for cancer drugs in the Chinese provincial-level centralized procurement process.

**Main Outcomes and Measures:**

The main outcome was the efficacy and safety associated with BTD vs non-BTD cancer drugs, including progression-free survival (PFS), response rate (RR), duration of response, serious adverse events, grade 3 or higher adverse events, and treatment-related deaths. In addition, the time to approval, novelty, and initial and latest average monthly treatment prices were evaluated, as well as the average annual reduction rate (AARR; the sum of the reduction rates divided by the number of years for the monthly treatment price) for these cancer drugs.

**Results:**

Between July 2020 and July 2024, 18 BTD (36%) and 32 non-BTD (64%) cancer drugs were approved by the NMPA. The median (IQR) clinical development time for BTD drugs was significantly shorter than for non-BTD drugs (5.6 [95% CI, 4.3-7.3] vs 6.6 [95% CI, 6.0-8.5] years; *P* = .02). No significant differences were observed in PFS (HR, 0.44 [95% CI, 0.38-0.52] vs HR, 0.51 [95% CI, 0.40-0.65]; *P* = .20), PFS gained (median [IQR], 5.4 [3.9-7.0] vs 2.7 [2.6-5.9] months; *P* = .77), RR (58% [95% CI, 45%-74%] vs 59% [95% CI, 51%-69%]; *P* = .85), and duration of response (median [IQR], 18.0 [15.0-21.6] vs 11.1 [7.4-17.4] months; *P* = .09) between BTD and non-BTD drugs. The rates of serious adverse events (37% [95% CI, 26%-52%] vs 32% [95% CI, 27%-36%]; *P* = .45), adverse events grade 3 or higher (64% [95% CI, 53%-77%] vs 55% [95% CI, 45%-68%]; *P* = .31), and treatment-related deaths (2% [95% CI, 1%-4%] vs 1% [95% CI, 1%-2%]; *P* = .10) were similar between BTD and non-BTD drugs. BTD drugs are more likely to be first-in-class drugs (5 of 18 [28%] vs 1 of 32 [3%]; *P* = .02). Differences in the median (IQR) initial ($5665 [$3542-$9321] vs $3361 [$2604-$5474]; *P* = .06) and latest ($5665 [$1553-$9321] vs $2145 [$1318-$4276]; *P* = .18) average monthly treatment prices for BTD drugs and non-BTD drugs were not significant. The median (IQR) AARRs for BTD drugs and non-BTD drugs were 15.2% (0%-46.9%) and 19.8% (1.0%-42.9%), respectively.

**Conclusions and Relevance:**

The findings of this cross-sectional study suggest that BTD has facilitated faster time to market for cancer drugs and improved novelty, but the price of treatment is relatively higher. There was no significant difference on comparative efficacy and safety.

## Key Points

**Question:**

What are the differences in clinical benefit, clinical development time, novelty, and price of cancer drugs for breakthrough therapy designation (BTD) vs non-BTD cancer drugs in China?

**Findings:**

In a cross-sectional analysis of 18 BTD drugs and 32 non-BTD drugs, BTD drugs had significantly shorter clinical development times and higher novelty. There was no significant difference in efficacy, safety, and change in therapeutic prices.

**Meaning:**

These results suggest that the implementation of the BTD policy in China has expedited the availability of medicines for patients with cancer and is conducive to encouraging innovation in drug development with comparable clinical benefits; however, further efforts are still needed to improve the affordability of BTD drugs.

## Teaser

This cross-sectional study examines the implementation of a breakthrough therapy designation policy for cancer drugs in China based on clinical benefit, clinical development time, novelty, and price.

## Introduction

Drug development involves a lengthy process usually taking an average of 7 years.^1^ To accelerate drug development for a serious or life-threatening disease, the US Food and Drug Administration (FDA) first established the breakthrough therapy designation (BTD) in 2012.^1,2^ Subsequently, Japan and Europe established similar expedited pathways named SAKIGAKE and PRIME in 2015 and 2016, respectively. In 2020, China National Medical Products Administration (NMPA) also established a BTD, which allows qualifying drugs to be prioritized for clinical trial development guidance and rolling filings.^3^ Although the criteria for this accelerated program vary somewhat across agencies, they all emphasize the significant therapeutic advantages over existing therapies (eTable 1 in Supplement 1).

The majority of BTD drugs have been granted to novel cancer drugs in China during the last 4 years of this expedited policy, which is similar to the US.^4,5,6^ The previous study reported that 51 of 78 BTDs (65.4%) were granted for novel cancer drugs by the NMPA, attributable to the fact that China faces the highest in the number of deaths and new cases of cancer in the world.^7,8^ It should be noted that there has been debate recently as to whether BTD cancer drugs offer higher clinical value than non-BTD cancer drugs in the US.^5,6,9,10^ As its name implies, the drugs granted BTD are usually perceived by the general public as showing higher clinical benefit than a similarly effective alternative.^11,12,13^ However, previous studies have suggested that FDA-approved BTD drugs may overestimate the benefits and strength of the drugs with higher prices.^5,14^ Therefore, understanding the clinical benefits and price of BTD drugs can help physicians and patients develop better treatment options.

As a growing number of BTD cancer drugs are approved in China, their clinical benefits and prices have also attracted considerable attention. This study was designed to assess the differences between NMPA-approved BTD and non-BTD cancer drugs regarding the clinical benefit, defined as efficacy and safety, novelty, clinical development time, and monthly treatment costs, with a view to informing policy optimization.

## Methods

This cross-sectional study followed the relevant portions of Strengthening the Reporting of Observational Studies in Epidemiology (STROBE) reporting guidelines. Ethical approval and informed consent were not considered necessary in accordance with the requirements of the Biomedical Ethics Committee of Peking University People's Hospital because this research was based on publicly available data. The data sources involved in this study are shown in eTable 2 in Supplement 1.

### Cancer Drugs Identification

We identified new molecular entities approved by the NMPA for cancer drugs from the inception of BTD implementation (July 8, 2020) to July 8, 2024.^15^ Because the BTD was implemented to accelerate the development of novel drugs (defined as a drug that has not been marketed at home or abroad in China) and thus the novel drugs that have been marketed in foreign countries before July 8, 2020, were excluded. We analyzed the original indication without including supplementary indications, similar to the previous study.^16^ The drug@FDA database was searched to determine whether these cancer drugs were approved by the FDA or not.^17^ In addition, the novelty of these cancer drugs was classified into first-in-class drugs and non–first-in-class drugs based on whether they have novel pharmacological mechanisms according to what Lanthier et al^18^ reported. We determined whether these cancer drugs were eligible for public reimbursement based on the National Reimbursement Drug List (NRDL) issued in 2023. We extracted the marketing authorization holders, investigational new drug application, new drug application or biologics license application, application or license approval date, product type, and expedited pathways for these cancer drugs based on the predesigned criteria.

### Pivotal Trials Extraction

The pivotal trials for supporting the approval of cancer drugs were identified from the review report issued by the NMPA, the corresponding published peer-reviewed articles or other international meeting reports (eg, American Society of Clinical Oncology annual meeting). For randomized clinical trials (RCTs), we extracted hazard ratios (HRs) and median survival for overall survival (OS) and progression-free survival (PFS). For single-arm trials, we extracted the response rate (RR) and duration of response, which included both solid and hematological cancers. To assess the safety between BTD and non-BTD drugs, we extracted the treatment-emergent serious adverse events, treatment-emergent adverse events evaluated as grade 3 or higher, and treatment-related death. Improvement in OS by 2.5 months or PFS by 3 months for cancer drugs was considered as significant clinical benefit, consistent with Kumar et al.^19^

### Cancer Drug Price

We extracted the prices of initial (at launch) and latest (2024) winning bid prices for cancer drugs from provincial centralized procurement platforms, as described in a previous study.^20^ The average monthly treatment costs of cancer drugs were calculated based on a body weight of 70 kg or 1.7 m^2^ at the recommended dosage of the labeling published through the NMPA. To avoid the effect of inflation, the prices of cancer drugs were adjusted according to the annual consumption index published by the National Bureau of Statistics of China.^21^ In addition, we calculated the average annualized reduction rate (AARR), which is the sum of the reduction rates divided by the number of years for the monthly treatment price of these cancer drugs. Because it is not possible to determine the monthly treatment price of cellular therapies, they were not included in the price analysis. We converted to dollars at US $1 equal to Chinese ¥7.26. The detailed calculation of the price for cancer drugs can be found in previous studies.^8,20,22^

### Statistical Analysis

Categorical variables were shown as numbers (with percentages) and continuous variables were presented as medians (IQR). The Fisher exact test was used for categorical variables while the Mann-Whitney *U* test was used for continuous variables. Clinical development time (defined as the time from investigational new drug application date to new drug application or biologics license application approval date) was compared between BTD and non-BTD cancer drugs using Kaplan-Meier survival curves, log-rank tests, and Cox proportional hazards models, consistent with previous studies.^5,16^ For HR of PFS from RCT, DerSimonian-Laird random-effects meta-analysis with the inverse variance method was performed using the metagen function; for RR or safety outcomes from single-arm trials, Restricted Maximum Likelihood random-effects meta-analysis with inverse variance method was performed using the metaprop function. Cochran *Q* test was used to compare the differences of HR for PFS, RR and safety outcomes between BTD and non-BTD drugs. To assess the stability of the results, we conducted subgroup analyses of the primary efficacy end points, stratifying by drug types, therapy lines, cancer types, and marketing authorization holders. In addition, logistic regression was used to assess the association between the magnitude of RR and the probability of drugs receiving BTD. For some indicators not reported in the review report, labeling, or articles, we obtained them by contacting the developer or corresponding authors whenever possible. In addition, we compared the difference in missing values between BTD and non-BTD drugs. IBM SPSS version 20 (IBM Corporation) and R version 4.1.0 (R Statistical Computing Project) were performed for statistical analysis and graph generation. The R packages involved include meta (version 5.2.0), forestplot (version 1.10.1), and ggplot2 (version 3.4.0). Two-tailed *P* values less than .05 were considered statistically significant.

## Results

### Included Drug Characteristics

We included 50 novel cancer drugs with 18 (36%) BTD and 32 (64%) non-BTD drugs (eFigure in Supplement 1). The most common cancer types included lung cancer (18 drugs [36%]), followed by lymphoma (9 drugs [18.0%]) and myeloma (4 drugs [8.0%]) (eTable 3 in Supplement 1). For the 18 BTD drugs, 15 (83%) received conditional approval, and all of them required further confirmatory trials for postmarketing efficacy and safety assessment.

No significant differences were found for BTD vs non-BTD drugs concerning the marketing authorization holders, drug type, regulatory status of conditional approval, in or out of NRDL, cancer type, therapy line, and treatment type. However, chimeric antigen receptor T-cell therapies (4 of 18 [22%] vs 0; *P* = .02) were more likely to receive BTD (eTable 3 in Supplement 1). In addition, BTD drugs were more likely to be combined with priority review (17 of 18 [94%] vs 16 of 32 [50%]; *P* = .002) and conditional approvals (15 of 18 [83%] vs 16 of 32 [50%]; *P* = .03).

### Clinical Development Time

**Figure 1.**
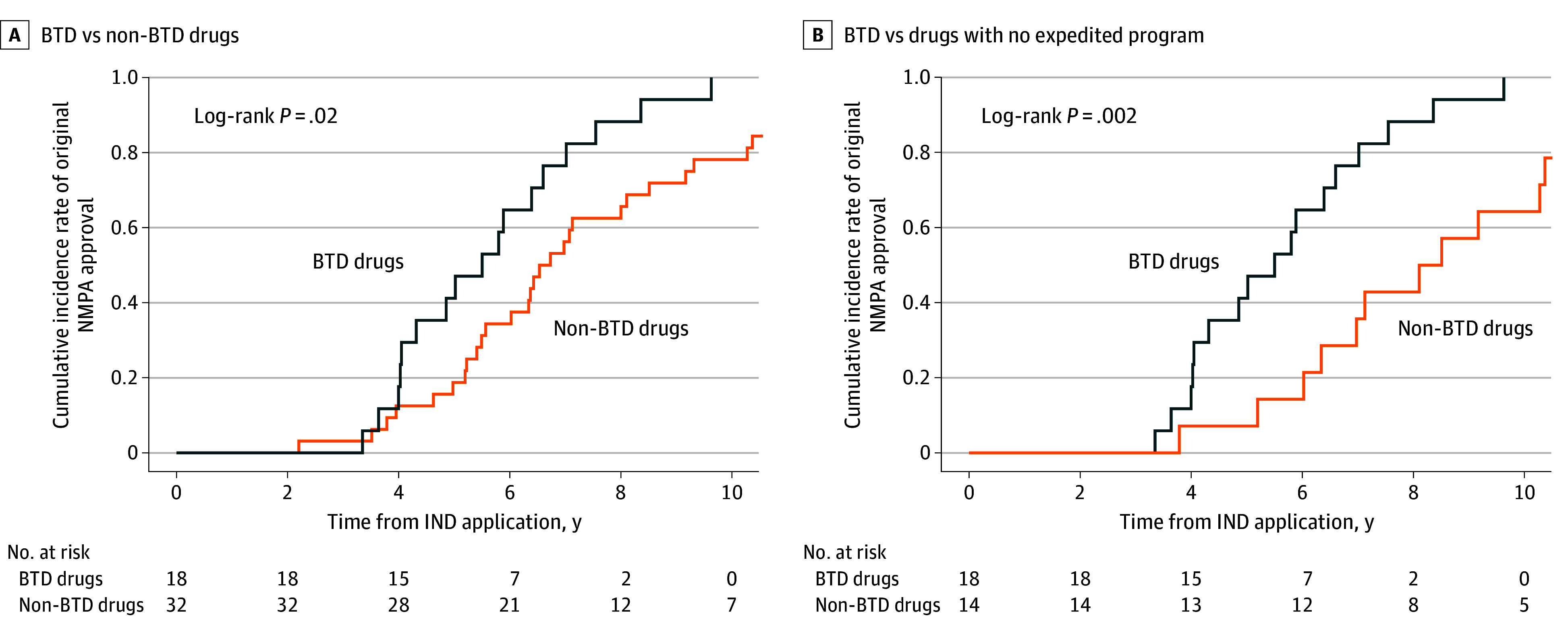
Time From Investigational New Drug Application to Original National Medical Products Administration (NMPA) Approval for Novel Cancer Drugs (A) Cumulative incidence rate of original NMPA approval for BTD (blue curve) vs no any expedited program (CA, BTD and PR) drugs (gold curve). (B) Cumulative incidence rate of original NMPA approval for BTD (blue curve) vs non-BTD drugs (gold curve). BTD indicates breakthrough therapy designation; CA, conditional approval; IND, investigational new drug; PR, priority review.

The development time of clinical trials for different subgroups of cancer drugs is shown in Table 1. The median (IQR) development time of clinical trials for imported drugs was less than that of domestic drugs by about 2.4 years (4.0 [3.6-6.8] vs 6.4 [5.2-8.2] years; *P* = .12), although these results were not significant. The results of both the log-rank test and the Cox proportional hazards models showed that BTD drugs had significantly shorter median clinical development times than drugs without BTD (5.6 [95% CI, 4.3-7.3] vs 6.6 [95% CI, 6.0-8.5] years, *P* = .02; HR, 2.0 [95% CI, 1.1-3.8]; *P* = .02) (Figure 1A) or did not receive any accelerated programs (5.6 [95% CI, 4.3-7.3] vs 8.3 [95% CI, 7.0-12.8] years; *P* = .002; HR, 3.5 [95% CI, 1.5-8.1]; *P* = .003) (Figure 1B).

**Table 1.** Time From Investigational New Drug (IND) Application to the China National Drug Administration Approval for Cancer Drugs, 2020-2024

Characteristic	Drugs, No. (%)	Time from IND to approval, median (IQR), y	*P* value^a^
Overall	50 (100)	6.4 (5.0-8.1)	NA
Market authorization			
Imported drugs	7 (14)	4.0 (3.6-6.8)	.12
Domestic drugs	43 (86)	6.4 (5.2-8.2)	
Cancer type			
Solid	36 (72)	6.7 (5.4-8.4)	.07
Blood	14 (28)	5.4 (4.0-6.4)	
Conditional approval			
CA with BTD	15 (30)	5.5 (4.2-6.8)	.91
CA with no BTD	16 (33)	5.5 (4.9-6.7)	
Any expedited program			
Yes	36 (72)	5.7 (4.5-7.0)	.005
No	14 (28)	8.3 (6.5-10.3)	
BTD			
BTD with CA	15 (30)	5.5 (4.2-6.8)	.95
BTD with no CA	3 (6)	5.8 (4.9-6.7)	
Expedited program^b^			
All BTD	18 (36)	5.6 (4.1-6.9)	.006
All PR	33 (66)	5.8 (4.6-7.1)	.007
All CA	31 (62)	5.5 (4.5-6.7)	.002
Combinations of programs^b^			
BTD with CA	15 (30)	5.5 (4.2-6.8)	.008
PR with CA	28 (56)	5.5 (4.5-6.8)	.003
PR with BTD	17 (34)	5.5 (4.0-7.0)	.005
PR, BTD, and CA	14 (28)	5.3 (4.1-6.9)	.008

Abbreviations: BTD, breakthrough therapy designation; CA, conditional approval; NA, not applicable; PR, priority review.

^a^ *P* values were calculated via the Mann-Whitney *U* test.

^b^ Some cancer drugs may be granted multiple expedited programs. *P* value was calculated based on comparisons with cancer drugs that did not receive any expedited program.

### Pivotal Trial Characteristics

The primary efficacy end points included RR (36 drugs [72%]), PFS (9 [18%]), OS (2 [4%]), or PFS and OS as co-primary end points (3 [6%]) (Table 2). A total of 34 cancer drugs (68%) were based on a single-arm trial while 16 (32%) were RCTs (eTable 4 in Supplement 1). The median (IQR) number of patients included in pivotal clinical trials supporting BTD drugs was significantly smaller compared with non-BTD drugs (104 [84-144] vs 209 [105-357]; *P* = .03), while no significant differences were seen in other variables (eTable 3 in Supplement 1).

**Table 2.** Comparison of the NMPA-Approved BTD and Non-BTD Cancer Drugs in Regard to the Primary Efficacy End Points, Safety, and Novelty

End point or outcome	Drugs, No. (%)		*P* values
	BTD drugs (n = 18)	Non-BTD drugs (n = 32)	
Primary efficacy end points, No. (%)			
RR for SAT	15 (83.3)	19 (59.4)	.53
RR for RCT	0	2 (6.3)	
PFS	2 (11.1)	7 (21.9)	
OS	0	2 (6.3)	
PFS and OS	1 (6.0)	2 (6.3)	
Efficacy			
RR, %^a^			
Median (IQR)	66 (42-79)	61 (48-78)	.82
Pooled estimate (95% CI)^b^	58 (45-74)	59 (51-69)	.85
Duration of response, median (IQR), mo^a^	18.0 (15.0-21.6)	11.1 (7.4-17.4)	.09
PFS			
Gain, median (IQR), mo	5.4 (3.9-7.0)	2.8 (2.6-5.9)	NA^c^
Pooled hazard ratio (95% CI)^b^	0.44 (0.38-0.52)	0.51 (0.40-0.65)	.32
Clinically meaningful improvement, No./total No. (%)^d^	1/2 (50.0)	2/7 (28.6)	>.99
Safety^e^			
Grade ≥3 AE, No. patients/total patients (%)	660/1063 (62.1)	899/1622 (55.4)	.39
Grade ≥3 AE, pooled estimate (95% CI)^b^	0.64 (0.53-0.77)	0.55 (0.45-0.68)	.31
SAE, No. patients/total patients (%)	337/859 (39.2)	319/1035 (30.8)	.56
SAE, pooled estimate (95% CI)^b^	0.37 (0.26-0.52)	0.32 (0.27-0.36)	.45
Treatment-related deaths, No. patients/total patients (%)	12/1155 (1.0)	9/1589 (0.5)	.26
Treatment-related deaths, pooled estimate (95% CI)^b^	0.02 (0.01-0.04)	0.01 (0.01-0.02)	.10
Novelty			
First-in-class, No./total No. (%)	5/18 (28)	1/32 (3)	.02

Abbreviations: AE, adverse event; BTD, breakthrough therapy designation; NA, not available; NMPA, National Medical Products Administration; OS, overall survival; PFS, progression-free survival; RCT, randomized clinical trial; RR, response rate; SAE, serious adverse event; SAT, single-arm trial.

^a^ Response rates and duration of response were derived from cancer drugs with single-arm trials, which included both solid and hematologic cancers. For solid cancers, response rates included partial and complete responses.

^b^ Results were from the meta-analysis.

^c^ PFS was derived from cancer drugs with randomized controlled trials. The statistical tests were not performed because only 2 BTD drugs reported PFS gains.

^d^ The median PFS for one of the cancer drugs was not matured.

^e^ Safety outcomes were derived from cancer drugs with single-arm design.

### Efficacy, Safety, and Novelty

**Figure 2.**
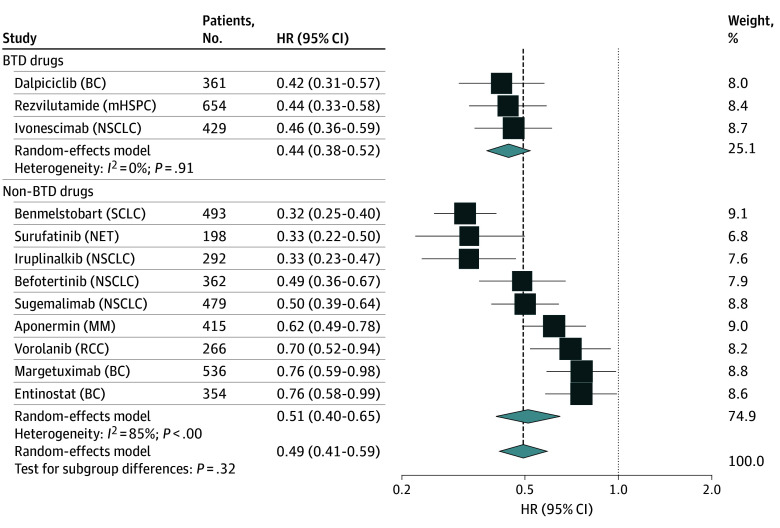
Forest Plot of PFS as Primary Efficacy End Points Derived From RCTs for BTD vs Non-BTD Cancer Drugs vs Placebo Confidence intervals for the RR were calculated using the Clopper-Pearson method and the variance of the pooled estimates of the proportions were stabilized using a logarithmic transformation. Vertical dashed lines indicate the results of the pooled assessment for all BTD and non-BTD drugs. BC indicates breast cancer; BTD, breakthrough therapy designation; HR, hazard ratio; mHSPC, metastatic hormone-sensitive prostate cancer; NET, neuroendocrine tumors; NSCLC, non-small cell lung cancer; PFS, progression-free survival; RCC, renal cell carcinoma; RCT, randomized clinical trial; RR, response rate; SCLC, small cell lung cancer.

No significant difference in the proportion of missing values was observed between BTD and non-BTD drugs (eTable 5 in Supplement 1). For efficacy, no significant difference was observed in the median (IQR) PFS gains (5.4 [3.9-7.0] vs 2.8 [2.6-5.9] months; *P* = .77), median (IQR) RR (66% [42%-79%] vs 61% [48%-78%]; *P* = .82), and median (IQR) duration of response (18.0 [15.0-21.6] vs 11.1 [7.4-17.4] months; *P* = .09) for BTD vs non-BTD drugs (Table 2). Similarly, the results from pooled analysis showed that no significant difference for HR for PFS (pooled HR: 0.44 [95% CI, 0.38-0.52] vs 0.51 [95% CI, 0.40-0.65]; *P* = .32) and RR (pooled estimate: 58% [95% CI, 45%-74%] vs 59% [95% CI, 51%-69%]; *P* = .85) was found between BTD and non-BTD drugs (Figure 2; eFigure 2 in Supplement 1). Subgroup analyses showed that no significant difference in RR between BTD and non-BTD drugs was observed (eTable 6 in Supplement 1). In addition, logistic regression indicated that the magnitude of the RR was not significantly related to BTD drugs (OR, 1.24 [95% CI, 0.05-30.36]; *P* = .90). For safety, there were no significant differences in the rates of serious adverse events (337 of 859 [39.2%] vs 319 of 1035 [30.8%]; *P* = .56), adverse events of grade 3 or higher (660 of 1063 [62.1%] vs 899 of 1622 [55.4%]; *P* = .39) or treatment-related deaths (12 of 1155 [1.0%] vs 9 of 1589 [0.5%]; *P* = .26) derived from single-arm trials for BTD vs non-BTD drugs. Consistently, the results of the pooled analysis supported this result. For novelty, the proportion of BTD drugs with first-in-class was significantly higher compared with non-BTD drugs (5 of 18 [28%] vs 1 of 32 [3%]; *P* = .02).

### Price of Cancer Drugs

**Figure 3.**
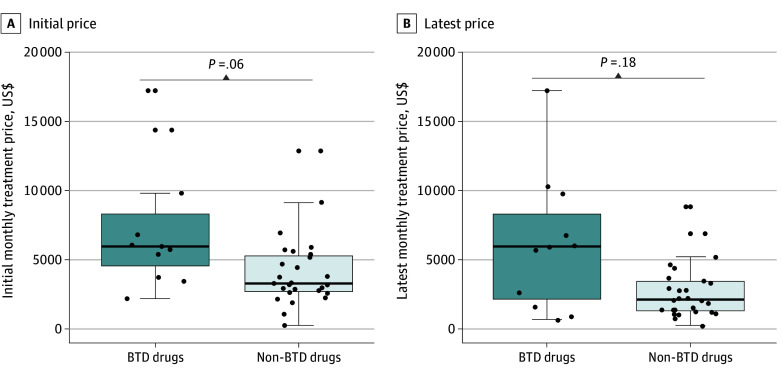
Monthly Treatment Price for Novel Cancer Drugs (A) Initial monthly treatment price for BTD vs non-BTD drugs. (B) The latest monthly treatment price for BTD vs non-BTD drugs. The area around the middle of the box represents the median, while the bottom and top edges of the box correspond to the first quartile (25th percentile) and the third quartile (75th percentile), respectively. The solid lines extending from the bottom and top of the box denote the lower and upper whiskers. BTD indicates breakthrough therapy designation.

The median (IQR) monthly treatment price was numerically higher for BTD drugs than for non-BTD drugs at initial price ($5665 [$3542-$9321] vs $3361 [$2604-$5474]; *P* = .06) and latest price ($5665 [$1553-$9321] vs $2145 [$1318-$4276]; *P* = .18), although these results were not statistically significantly (Figure 3). Significant reductions in median latest monthly treatment price were observed for non-BTD drugs compared with the initial price ($2145 vs $3361; *P* = .03); however, no significant differences were observed for BTD drugs ($5565 vs $5565; *P* = .62). From 2021 to 2024, the median AARR for monthly treatment price for BTD and non-BTD drugs was 15.2% and 19.8%, respectively.

## Discussion

Our findings indicated that the BTD and non-BTD drugs were comparable in their clinical benefits, similar to those previously reported by Hwang et al,^16^ which demonstrated no significant differences in efficacy and safety between FDA-approved BTD and non-BTD therapies. However, a 2024 study reported by Michaeli et al^5^ demonstrated that the FDA-approved BTD drugs were more likely to improve higher clinical benefit than non-BTD drugs. The differences that contributed to the results of the 2 studies may be related to the sample included and the range of drugs. It should be noted that the study reported by Michaeli et al^5^ included not only the original indication but also the supplemental indications. Typically, the original indication is for patients with cancer who are in end-line therapy or have no available therapy, so it may be more difficult to distinguish the clinical value between BTD and non-BTD drugs. In this study, we included only the original indication with limited samples. Therefore, these findings remain to be verified by expanding the sample (eg, including the supplement indications) in the future.

Interestingly, it was noted that the pooled analysis of the HR for PFS in China for BTD drugs was comparable with the FDA-approved BTD drugs (0.43 vs 0.42). Similarly, the pooled RR for solid cancer from single-arm trials indicated comparable outcomes for cancer drugs with BTD in China and the US (45% vs 37%).^5^ This evidence suggests that cancer drugs granted BTD in China have similar efficacy requirements compared with BTD in the US. In addition, we found that the majority of approvals for BTD drugs and non-BTD drugs were based on surrogate end points, similar to the results in the US.^16^ This partly explains why 31 cancer drugs (62.0%) received conditional approval, requiring postmarket confirmatory trials to determine if they should gain full approval or be withdrawn. Therefore, as previous studies suggest, the clinical benefit of BTD vs non-BTD drugs should also consider confirmatory trial evidence, particularly regarding OS and quality of life.^23,24,25^

Our results showed that the median clinical development time for BTD cancer drugs in China was 5.6 years, similar to that of the FDA (median length of 5.2 years).^16^ BTD drugs were 1 year shorter than non-BTD drugs and 2.7 years shorter than without any expedited program approvals, suggesting that the implementation of the BTD policy in China has significantly facilitated drug development for serious diseases. It should be noted that BTD drugs enrolled significantly fewer patients than non-BTD drugs similar to the result of the FDA, which may be an important factor in the faster time to market for BTD drugs.^5^ Previous studies have shown that smaller trials are less likely to observe rare adverse events and may also lead to biased results in measuring efficacy.^26,27^ Therefore, the NMPA should enhance postmarketing monitoring of the efficacy and safety of these BTD drugs.

It is well known that China has greatly promoted the development of novel drugs since the reform of drug regulation and review in 2015.^28,29,30^ However, the phenomenon of duplication of research and development for some drug targets has also emerged, resulting in a waste of clinical resources, especially in the field of cancer.^31^ In addition, the majority of the novel drugs developed locally in China are follow-on products and lack first-in-class drugs.^8^ Therefore, the NMPA released the Clinical Value-Driven Clinical Development Guidelines for Cancer Drugs in 2021, which emphasizes the significance of original innovation.^32^ This study found that BTD drugs had a higher proportion of first-in-class drugs compared with non-BTD drugs, suggesting that the BTD policy supports the development of novel drugs in China.

The high burden of oncology treatment has been a global concern, severely affecting the accessibility of medicines for patients.^33,34^ The previous study showed that FDA-approved BTD drugs had higher average monthly treatment prices than non-BTD drugs.^5^ In this study, higher monthly treatment prices for BTD drugs than non-BTD drugs were observed, although these results were not significant. This suggests that attention should be paid to the affordability of BTD drugs, with an emphasis on not letting the BTD designation drive costs to be excessively high, considering that there is no significant difference in their clinical benefit. Additionally, we found that the AARR of BTD and non-BTD drugs were 15% and 20% respectively, indicating a decreasing trend in treatment costs over years for both BTD and non-BTD drugs, which is in contrast to the increase annually in the US.^5^ This result is primarily attributed to national drug negotiations established in China since 2016.^8,35,36^ It should be admitted that the median monthly treatment prices for the latest (2024) BTD drugs estimated in this study was $5665, which was more than 10 times the per capita disposable income ($476 per month) according to data reported by the China National Statistical Office in 2024.^37^ Therefore, these BTD drugs in the absence of NRDL inclusion would be a catastrophic expense for most families.

### Limitations

This study has some limitations. First, the BTD policy in China was officially implemented in July 2020, so its duration has been relatively short, and the sample included is also limited. Second, this study focused on the original indication and did not consider supplemental indications, which is similar to the previous study.^16^ The original indication is a better representation of the availability of medication to the patient than the supplemental indications. Third, in this study, 32 cancer drugs received conditional approval based on preliminary trial evidence and their risk-benefit relationships may change as a result of confirmatory trials. Because the majority of confirmatory trials are still ongoing, this part of the evidence was not evaluated in this study. Fourth, the clinical development time was shorter in conditional approval (compared with regular approval) and in imported drugs (compared with domestic drugs) may be confounded to each other. It should be admitted that the conditional approval for imported drugs may be based on global RCT and local single-arm trials in condition of postapproval commitment study. In addition, imported drugs may have already completed phase 1 or phase 2 trials by the time they submit an investigational new drug application in China. Therefore, these factors may have combined to result in imported drugs taking less time to develop trials in China. Fifth, there was significant heterogeneity when combining different cancer types, although no significant difference between the cancer types of BTD and non-BTD drugs included. Ideally, it might be preferable to do analyses stratified by cancer type, but the small number of drugs we studied precludes that possibility. Finally, in this study, certain items were missing due to underreporting, which may introduce bias into the results, although similar data gaps were present for both BTD and non-BTD drugs.

## Conclusions

The implementation of BTD policy in China has successfully expedited the market entry of drugs for cancer with limited treatment options, while also increasing the proportion of first-in-class drugs, reflecting support for and encouragement of original innovation. The efficacy and safety between BTD drugs and non-BTD drugs were comparable. However, there was some evidence that treatment prices of BTD drugs were relatively higher. As the majority of cancer drugs are granted conditional approval, further evaluation including OS and quality of life or safety evidence is needed to verify whether BTD drugs have added clinical benefits.

## Notes

Supplement 1. **eTable 1.** Characteristics of Breakthrough Therapies and Other Similar Accelerated Pathways for Novel Drug Development **eTable 2.** Data Source **eTable 3.** Comparison of Characteristics of the NMPA-Approved BTD and Non-BTD Drugs **eTable 4.** Characteristics of Pivotal Trials for BTD and Non-BTD drugs **eTable 5.** Comparison of BTD and Non-BTD Drugs in the Proportion of Missing Values **eTable 6.** Subgroup Analysis for Comparison of Response Rates of BTD Drugs and Non-BTD Drugs by Meta-analysis **eFigure 1.** Flow Chart of the Included Cancer Drugs in the Analysis **eFigure 2.** Forest Plot of RR as Primary Efficacy End Points Derived From Single-Arm Trials for BTD vs Non-BTD Cancer Drugs
eReferences.

Supplement 2.
Data Sharing Statement
