# Supplementary material for: Clinical Benefit, Price, and Regulatory Approval of Cancer Drugs Granted Breakthrough Therapy Designation in China, 2020-2024
Source: JAMA Netw Open. 2024 Oct 16;7(10):e2439080. doi: 10.1001/jamanetworkopen.2024.39080 (PMC11581593; doi:10.1001/jamanetworkopen.2024.39080)
Supplement: Supplement 1. — eTable 1. Characteristics of Breakthrough Therapies and Other Similar Accelerated Pathways for Novel Drug Development eTable 2. Data Source eTable 3. Comparison of Characteristics of the NMPA-Approved BTD and Non-BTD Drugs eTable 4. Characteristics of Pivotal Trials for BTD and Non-BTD drugs eTable 5. Comparison of BTD and Non-BTD Drugs in the Proportion of Missing Values eTable 6. Subgroup Analysis for Comparison of Response Rates of BTD Drugs and Non-BTD Drugs by Meta-analysis eFigure 1. Flow Chart of the Included Cancer Drugs in the Analysis eFigure 2. Forest Plot of RR as Primary Efficacy End Points Derived From Single-Arm Trials for BTD vs Non-BTD Cancer Drugs eReferences. [file jamanetwopen-e2439080-s001.pdf]

Supplemental Online Content

Luo X, Du X, Lv X, Yang Y, Zhang X, Huang L. Clinical benefit, price and regulatory approval of cancer drugs granted breakthrough therapy designation in China, 2020-2024. *JAMA Netw Open.* 2024;7(10):e2439080. doi:10.1001/jamanetworkopen.2024.39080

eTable 1. Characteristics of Breakthrough Therapies and Other Similar Accelerated Pathways for Novel Drug Development

eTable 2. Data Source

eTable 3. Comparison of Characteristics of the NMPA-Approved BTD and Non-BTD Drugs

eTable 4. Characteristics of Pivotal Trials for BTD and Non-BTD drugs

eTable 5. Comparison of BTD and Non-BTD Drugs in the Proportion of Missing Values

eTable 6. Subgroup Analysis for Comparison of Response Rates of BTD Drugs and Non-BTD Drugs by Meta-analysis

eFigure 1. Flow Chart of the Included Cancer Drugs in the Analysis

eFigure 2. Forest Plot of RR as Primary Efficacy End Points Derived From Single-Arm Trials for BTD vs Non-BTD Cancer Drugs

eReferences.

This supplemental material has been provided by the authors to give readers additional information about their work.

© 2024 Luo X et al. *JAMA Network Open.*

eTable 1. Characteristics of breakthrough therapies and other similar accelerated pathways for novel drug development.

Items	The US	Japan	Europe	China
Names	Breakthrough Therapy	SAKIGAKE	PRIME	Breakthrough Therapy
Regulatory agency	FDA	PMDA	EMA	NMPA
Effective period	2012–present	2015-–present	2016–present	2020–present
When to submit	With IND or after;<br>Ideally, no later than the end-of-phase 2 meeting	By sponsor at prespecified application dates	Any sponsor engaged in the exploratory clinical trial phase of development	Phase I and II clinical trials; Ideally, no later than Phase III
Designation criteria	Alone or in combination with 1 or more other drugs to treat a serious or life-threatening disease or condition; Preliminary clinical evidence indicates that the drug may demonstrate substantial improvement over existing therapies on 1 or more clinically significant endpoints.	Having developed firstly in Japan and anticipating an application for approvals (desirable to have PMDA consultation from the beginning of R&D);<br>Prominent effectiveness (i.e. radical improvement compared to existing therapy), can be expected based on the data of mechanism of action from non-clinical study and early phase of clinical trials (phase I to II).	Must address an unmet medical need, that is, for which there exists no satisfactory treatment;<br>Brings major therapeutic advantages to those affected; Preliminary clinical data must be available.	Novel drugs or modified novel drugs (defined as not marketed at home or abroad);<br>For serious or life-threatening diseases;<br>For serious impact on quality of life; Preliminary clinical evidence that suggests substantial improvement over existing therapies;<br>Clinical trial data should include population in China;
Benefits of the expedited program	Intensive Guidance on an Efficient Drug Development Program, Beginning as Early as Phase 1;<br>Organizational Commitment; Involving Senior Managers; Submission of Portions of an Application (Rolling Review).	Prioritized consultation by the PMDA; Substantial pre-application consultation;<br>Prioritized review of the market application;<br>Review concierge assigned by PMDA;<br>Extension of re-examination period as well as facilitating coalition with scientific societies, and transmission of information globally.	Early appointment of CHMP or CAT rapporteur;<br>Kick-off meeting with the rapporteur and multidisciplinary group of experts from EMA / European medicines regulatory network;<br>Appointment of PRIME Scientific Coordinator;<br>Iterative scientific advice on overall development plans and key issues;<br>Expedited follow-up scientific advice;<br>Submission readiness meeting;<br>Confirmation of potential accelerated assessment.	Prioritized consultation by the NMPA;<br>Eligible for priority review and conditional approval;<br>Rolling submission;<br>Intensive guidance from regulatory agencies;
Timelines for response	Within 60 calendar days of receipt of the request	Application is to be submitted to Evaluation and Licensing Division and will be notified within 60 days.	EMA updates the list of all products granted access to the PRIME scheme below every month.	Within 45 working days of receipt of the request
Data source	https://www.fda.gov/patients/fast-track-breakthrough-therapy-accelerated-approval-priority-review/breakthrough-therapy	https://www.mhlw.go.jp/english/policy/health-medical/pharmaceuticals/140729-01.html	https://www.ema.europa.eu/en/human-regulatory-overview/research-development/prime-priority-medicines#key-benefits-for-applicants-12631	https://www.nmpa.gov.cn/xxgk/ggtg/ypggtg/ypqtggtg/20200708151701834.html.

Abbreviations: NMPA, National Medical Products Administration; FDA, US Food and Drug Administration; PMDA, Pharmaceuticals and Medical Devices Agency; EMA, European Medicines Agency; IND, investigational new drug.

© 2024 Luo X et al. *JAMA Network Open.*

eTable 2. Data Source.

Source	Variable	Website
NMPA	Priority review	https://www.cde.org.cn/main/xxgk/listpage/2f78f372d351c6851af7431c7710a731
	Conditional approval	
	Breakthrough therapy designation	
	Review report	
	IND application date	
	NDA/BLA approval date	
National Healthcare Security Administration	National Reimbursement Drug List (2023)	https://www.nhsa.gov.cn/art/2023/12/13/art_14_11671.html
Clinical Trial Website	NCT number	https://clinicaltrials.gov/
	CTR number	http://www.chinadrugtrials.org.cn/index.html
National Bureau of Statistics	Per capita disposable income	https://www.stats.gov.cn/sj/zxfb/202407/t20240715_1955615.html
	CPI inflation	https://data.stats.gov.cn/easyquery.htm?cn=C01
US FDA	NDA/BLA approval date	https://www.accessdata.fda.gov/scripts/cder/daf/index.cfm
Provincial centralized procurement platforms	Winning bid price for cancer drugs	http://www.ynyyzb.com.cn/detail.html?infoId=22112&CatalogId=3 (example)
Drug Bank	Mechanism of action and product type	https://go.drugbank.com/

Abbreviations: CPI, consumer price index; NMPA, National Medical Products Administration; FDA, US Food and Drug Administration; NDA, new drug application; BLA, biologics license application; IND, investigational new drug.

© 2024 Luo X et al. *JAMA Network Open.*

eTable 3. Comparison of characteristics of the NMPA-approved BTD and non-BTD drugs.

Characteristics	Overall（n=50) No. (%)	BTD Drugs (n=18) No. (%)	No BTD Drugs (n = 32) No. (%)	*P* values
**Drug characteristics**				
MAH				
Domestic	43 (86.0)	14 (77.8)	29 (90.6)	.23
Imported	7 (14.0)	4 (22.2)	3 (9.4)	
Drug types				
NDA	27 (54.0)	9 (50.0)	18 (56.3)	.77
BLA	23 (46.0)	9 (50.0)	14 (43.7)	
Product type				
Small-molecule	27 (54.0)	9 (50.0)	18 (56.3)	.02
Antibody	19 (38.0)	5 (27.8)	14 (44.8)	
Cell therapy	4 (8.0)	4 (22.2)	0 (0)	
NRDL (2023)				
Included	19 (38.0)	4 (22.2)	15 (46.9)	.13
Not included	31 (62.0)	14 (77.8)	17 (53.1)	
**NMPA expedited programs**				
Priority review				
Yes	33 (66.0)	17 (94.4)	16 (50.0)	.002
No	17 (34.0)	1 (5.6)	16 (50.0)	
Conditional approval				
Yes	31 (62.0)	15 (83.3)	16 (50.0)	.03
No	19 (38.0)	3 (16.7)	16 (50.0)	
Regulatory status of CA				
Ongoing	24 (48.0)	13 (72.2)	11 (34.4)	.40
Full approval	5 (10.0)	1 (5.6)	4 (12.5)	
Withdrawn	1 (2.0)	1 (5.6)	0 (0)	
Others	1 (2.0)	0 (0)	1 (3.1)	
**Indication characteristics**				
Cancer types				
Lung cancer	18 (36.0)	6 (33.3)	12 (37.5)	.08
Lymphoma	9 (18.0)	4 (22.2)	5 (15.6)	
Myeloma	4 (8.0)	3 (16.7)	1 (3.1)	
Breast cancer	3 (6.0)	1 (5.6)	2 (6.3)	
Ovarian cancer	2 (4.0)	0 (0)	2 (6.3)	
Solid cancer	2 (4.0)	0 (0)	2 (6.3)	
Gastric cancer	2 (4.0)	0 (0)	2 (6,3)	
Others	11 (22.0)	4 (22.2)	7 (21.9)	
Treatment types				
Combination	12 (24.0)	3 (16.7)	9 (28.1)	.50
Monotherapy	38 (76.0)	15 (83.3)	23 (71.8)	
Therapy lines				
First-line	14 (28.0)	4 (22.2)	10 (31.3)	.74
Later-line	36 (72.0)	14 (77.8)	22 (68.8)	
Biomarker status				
Yes	29 (58.0)	7 (38.9)	22 (69)	.07
No	21 (42.0)	11 (61.1)	10 (31)	

© 2024 Luo X et al. *JAMA Network Open.*

Characteristics	Overall（n=50）<br>No. (%)	BTD Drugs (n=18)<br>No. (%)	No BTD Drugs (n = 32)<br>No. (%)	*P* values
**Clinical trial characteristics**				
Enrolled patients, median (IQR)	113 (99, 338)	104 (84, 144)	209 (105, 357)	.03
Clinical trial phase				
I phase	2 (4.0)	1 (5.6)	1 (3.1)	.19
II phase	32 (64.0)	14 (77.8)	18 (56.3)	
III phase	16 (32.0)	3 (16.7)	13 (40.6)	
Trial design				
Single-arm	34 (68.0)	15 (83.3)	19 (59.4)	.12
RCT	16 (32.0)	3 (16.7)	13 (40.6)	
MRCT				
Yes	9 (18.0)	6 (33.3)	3 (9.4)	.06
No	41 (82.0)	12 (66.7)	29 (90.6)	
**Price of cancer drugs**				
Initial monthly treatment price ($), median (IQR)	4217 (2758, 6112)	5665 (3542, 9321)	3361 (2604, 5475)	.06
Latest monthly treatment price ($), median (IQR)	2679 (1352, 5710)	5665 (1553, 9321)	2145 (1318, 4276)	.18
Included in NRDL (2023)	1356 (1055, 2017)	1223 (711, 2287)	1356 (1094, 2017)	.55
Not included in NRDL (2023)	5557(2798, 9445)	6468 (5665, 9817)	4217 (2679, 8440)	.10

Abbreviations: MAH, market authorization holders; NMPA, National Medical Products Administration; FDA, US Food and Drug Administration; NDA, new drug application; BLA, biologics license application; IQR, interquartile range; BTD, breakthrough therapy designation; RCT, randomized controlled trial; MRCT, multi-regional clinical trial; NRDL, National Reimbursement Drug List.

© 2024 Luo X et al. *JAMA Network Open.*

eTable 4. Characteristics of pivotal trials for BTD and non-BTD drugs.

Generic name	Cancer types	First-in-class	NRDL (2023)	Therapy lines	Trial phase*	MAH	Drug types	NCT/CTR number	Trial name	Trial design	Primary efficacy endpoints[&]	MRCT[#]	Priority review	Conditional approval	Breakthrough therapy designation	Reference
Ensartinib	Lung cancer	No	Yes	Later-line	2	Domestic	NDA	NCT03215693	NA	Single-arm	RR	No	Yes	Yes	No	1,2
Fluzoparib	Ovarian cancer	No	Yes	Later-line	1	Domestic	NDA	NCT03509636	NA	Single-arm	RR	No	Yes	Yes	No	3
Orelabrutinib	Lymphoma	No	Yes	Later-line	2	Domestic	NDA	NCT03494179	ICP-CL-00102	Single-arm	RR	No	Yes	Yes	No	4
Surufatinib	Neuroendocrine tumor	No	Yes	First-line	3	Domestic	NDA	NCT02588170	SANET-ep	RCT	PFS	No	Yes	No	No	5
Pralsetinib	Lung cancer	No	No	Later-line	2	Imported	NDA	NCT03037385	ARROW	Single-arm	RR	Yes	Yes	Yes	No	6
Pamiparib	Ovarian cancer	No	Yes	Later-line	2	Domestic	NDA	NCT03333915	NA	Single-arm	RR	No	Yes	Yes	No	7
Disitamab	Gastric cancer	No	Yes	Later-line	2	Domestic	BLA	NCT03556345	NA	Single-arm	RR	No	Yes	Yes	No	8
Savolitinib	Lung cancer	No	Yes	Later-line	2	Domestic	NDA	NCT02897479	NA	Single-arm	RR	No	Yes	Yes	No	9
Penpulimab	Lymphoma	No	No	Later-line	2	Domestic	BLA	NCT03722147	AK105-201	Single-arm	RR	No	No	Yes	No	10
Zimberelimab	Lymphoma	No	No	Later-line	2	Domestic	BLA	NCT03655483	NA	Single-arm	RR	No	No	Yes	No	11
Relmacabtagene	Lymphoma	No	No	Later-line	2	Domestic	BLA	NCT04089215	RELIANCE	Single-arm	RR	No	Yes	Yes	Yes	12
Olverembatinib	Leukemia	No	Yes	Later-line	2	Domestic	NDA	NCT03883087；NCT03883100	HQP1351-CC-201；HQP1351-CC-202N	Single-arm	RR	No	Yes	Yes	No	13
Envafolimab	Gastric cancer	No	No	Later-line	2	Domestic	BLA	NCT03667170	KN035	Single-arm	RR	No	Yes	Yes	No	14
Sugemalimab	Lung cancer	No	No	First-line	3	Domestic	BLA	NCT03789604	GEMSTONE-302	RCT	PFS	No	No	No	No	15
Dalpiciclib	Breast cancer	No	Yes	Later-line	3	Domestic	NDA	NCT03927456	DAWNA-1	RCT	PFS	No	Yes	No	Yes	16
Serplulimab	Solid cancer	No	No	Later-line	2	Domestic	BLA	NCT03941574	NA	Single-arm	RR	No	Yes	Yes	No	17
Rezvilutamide	Prostate cancer	No	Yes	First-line	3	Domestic	NDA	NCT03520478	CHART	RCT	PFS and OS	No	Yes	Yes	Yes	18
Cadonilimab	Cervical cancer	Yes	No	Later-line	2	Domestic	BLA	NCT03852251	COMPASSION-03	Single-arm	RR	No	Yes	Yes	Yes	19
Furmonertinib	Lung cancer	No	Yes	Later-line	2	Domestic	NDA	NCT03452592	AST2818	Single-arm	RR	No	Yes	Yes	No	20
Pucotenlimab	Solid cancer	No	No	Later-line	2	Domestic	BLA	NCT03704246	NA	Single-arm	RR	No	Yes	Yes	No	21
Donafenib	Hepatocellular carcinoma	No	Yes	First-line	3	Domestic	NDA	NCT02645981	ZGDH3	RCT	OS	No	Yes	No	No	22
Linperlisib	Lymphoma	No	Yes	Later-line	2	Domestic	NDA	NCT04370405	NA	Single-arm	RR	No	Yes	Yes	Yes	23
Mobocertinib	Lung cancer	No	No	Later-line	2	Imported	NDA	NCT02716116	NA	Single-arm	RR	Yes	Yes	Yes	Yes	24,25
Adebrelimab	Lung cancer	No	No	First-line	3	Domestic	BLA	NCT03711305	CAPSTONE-1	RCT	OS	No	No	No	No	26
Glumetinib	Lung cancer	No	Yes	First-line	2	Domestic	NDA	NCT04270591	GLORY	Single-arm	RR	Yes	Yes	Yes	Yes	27
Befotertinib	Lung cancer	No	Yes	Later-line	3	Domestic	NDA	NCT04206072	iCROSS	RCT	PFS	No	No	No	No	28
Vorolanib	Renal cell carcinoma	No	Yes	Later-line	3	Domestic	NDA	NCT03095040	CONCEPT	RCT	PFS	No	No	No	No	29
Iruplinalkib	Lung cancer	No	Yes	Later-line	3	Domestic	NDA	NCT04632758	INSPIRE	RCT	PFS	No	No	No	No	30
Equecabtagene	Myeloma	No	No	Later-line	2	Domestic	BLA	NCT05066646	FUMANBA-1	Single-arm	RR	No	Yes	Yes	Yes	31

© 2024 Luo X et al. *JAMA Network Open.*

Generic name	Cancer types	First-in-class	NRDL (2023)	Therapy lines	Trial phase*	MAH	Drug types	NCT/CTR number	Trial name	Trial design	Primary efficacy endpoints&	MRCT#	Priority review	Conditional approval	Breakthrough therapy designation	Reference
Sunvozertinib	Lung cancer	Yes	No	Later-line	2	Domestic	NDA	NCT05712902	WU-KONG6	Single-arm	RR	No	Yes	Yes	Yes	32
Margetuximab	Breast cancer	No	No	Later-line	3	Imported	BLA	NCT02492711	SOPHIA	RCT	PFS and OS	Yes	No	No	No	33,34
Narlumosbart	Giant-cell tumor of bone	No	No	First-line	2	Domestic	BLA	NCT04255576	JMT103CN03	Single-arm	RR	No	Yes	Yes	No	35
Aponermin	Myeloma	Yes	No	Later-line	3	Domestic	BLA	CTR20140751	CPT-MM301	RCT	PFS	No	No	No	No	36
Inaticabtagene	Leukemia	No	No	Later-line	2	Domestic	BLA	NCT04684147	NA	Single-arm	RR	No	Yes	Yes	Yes	37
Glofitamab	Lymphoma	No	No	Later-line	2	Imported	BLA	NCT03075696	NA	Single-arm	RR	Yes	Yes	Yes	Yes	38
Vebreltinib	Lung cancer	No	No	First-line	2	Domestic	NDA	NCT04258033	KUNPENG	Single-arm	RR	No	Yes	Yes	Yes	39
Tepotinib	Lung cancer	No	No	First-line	2	Imported	NDA	NCT02864992	VISION	Single-arm	RR	Yes	No	No	No	40
Socazolimab	Cervical cancer	No	No	Later-line	1	Domestic	BLA	NCT03676959	NA	Single-arm	RR	No	No	Yes	Yes	41
Zevorcabtagene	Myeloma	No	No	Later-line	2	Domestic	BLA	NCT03975907	Lummicar-1	Single-arm	RR	No	Yes	No	Yes	42
Tunlametinib	Melanoma	No	No	Later-line	2	Domestic	NDA	NCT05217303	NA	Single-arm	RR	No	Yes	Yes	No	43
Unecritinib	Lung cancer	No	No	First-line	2	Domestic	NDA	NCT03019276; NCT03972189	NA	Single-arm	RR	No	No	No	No	44
Entinostat	Breast cancer	No	No	Later-line	3	Domestic	NDA	NCT03538171	NA	RCT	PFS	No	No	No	No	45
Benmelstobart	Lung cancer	No	No	First-line	3	Domestic	BLA	NCT04234607	ETER701	RCT	PFS and OS	No	No	No	No	46
Repotrectinib	Lung cancer	No	No	First-line	2	Imported	NDA	NCT03093116	TRIDENT-1	Single-arm	RR	Yes	Yes	Yes	Yes	47
Rezivertinib	Lung cancer	No	No	Later-line	2	Domestic	NDA	NCT03812809	NA	Single-arm	RR	No	No	No	No	48
Ivonescimab	Lung cancer	Yes	No	Later-line	3	Domestic	BLA	NCT05184712	HARMONi-A	RCT	PFS	Yes	Yes	No	Yes	49
Golidocitinib	Lymphoma	Yes	No	Later-line	2	Domestic	NDA	CTR20213318	NA	Single-arm	RR	No	Yes	Yes	Yes	50
Zuberitamab	Lymphoma	No	Yes	First-line	3	Domestic	BLA	CTR20180855	NA	RCT	RR	No	No	No	No	51
Ripertamab	Lymphoma	No	Yes	First-line	3	Domestic	BLA	NCT02772822	NA	RCT	RR	No	No	No	No	52
Teclistamab	Myeloma	Yes	No	Later-line	2	Imported	BLA	NCT03145181; NCT04557098	MajesTEC-1	Single-arm	RR	Yes	Yes	Yes	Yes	53,54

* In this study, labeled phase 1/2 trials were categorized as phase 2 while labeled phase 2/3 trials were categorized as phase 3. & RR includes both hematology and solid cancer. For solid tumors, RR includes partial and complete response. # MRCT defined as a clinical trial conducted in multiple countries. Abbreviations: MAH, market authorization holders; BTD, breakthrough therapy designation; RR, response rate; NDA, new drug application; BLA, biologics license application; IND, investigational new drug; RCT, randomized controlled trial; MRCT, multi-regional clinical trial; NRDL, National Reimbursement Drug List; NA, not available.

© 2024 Luo X et al. *JAMA Network Open.*

eTable 5. Comparison of BTD and non-BTD drugs in the proportion of missing values.

Variable		BTD drugs (n=18)		Non-BTD drugs (n=32)		*P* values
		Overall	No. of not reported (%)	Overall	No. of not reported (%)	
SAT	RR	15	0 (0.0)	19	0 (0.0)	NA
	DOR	15	7 (46.7)	19	8 (42.1)	1.00
	Grade≥3 AE	15	8 (53.3)	19	8 (42.1)	0.73
	SAE	15	10 (66.7)	19	11 (57.9)	0.50
	Treatment-related death	15	7 (46.7)	19	7 (36.8)	0.73
RCT	HR of PFS	3	0 (0.0)	9	0 (0.0)	NA
	Gains of PFS	3	1 (33.3)	9	2 (22.2)	1.00

**P* values were calculated by comparing the difference in missing values between BTD and non-BTD drugs using Fisher's exact test. Abbreviations: BTD, breakthrough therapy designation; SAT, single-arm trial; RCT, randomized controlled trial; RR, response rate; HR, hazard ratio; PFS, progression-free survival; DOR, duration of response; AE, adverse events; SAE, serious adverse events.

© 2024 Luo X et al. *JAMA Network Open.*

eTable 6. Subgroup analysis for comparison of response rates of BTD drugs and non-BTD drugs by meta-analysis.

Variables	Number of studies	Number of patients	Outcomes	*P*-value
			RR (95% CI)	
**Small-molecule drugs**				
BTD drugs	7	585	0.59 (0.45, 0.78)	.76
Non-BTD drugs	13	1644	0.62 (0.55, 0.71)	
**Biological product**				
BTD drugs	8	806	0.54 (0.38, 0.85)	.92
Non-BTD drugs	7	572	0.54 (0.38, 0.78)	
**Hematologic cancer**				
BTD drugs	8	788	0.70 (0.56, 0.88)	.20
Non-BTD drugs	4	327	0.85 (0.78, 0.92)	
**Solid cancer**				
BTD drugs	7	603	0.45 (0.29, 0.70)	.47
Non-BTD drugs	15	1888	0.54 (0.46, 0.63)	
**Lung cancer**				
BTD drugs	5	413	0.59 (0.42, 0.84)	.82
Non-BTD drugs	7	1165	0.62 (0.53, 0.71)	
**First-line therapy**				
BTD drugs	3	202	0.74 (0.66, 0.82)	.70
No BTD drugs	3	456	0.69 (0.52, 0.93)	
**Later-line therapy**				
BTD drugs	12	1189	0.53 (0.40, 0.73)	.75
No BTD drugs	16	1773	0.57 (0.48, 0.68)	
**Domestic drugs**				
BTD drugs	12	887	0.61 (0.45, 0.82)	.90
No BTD drugs	17	1816	0.60 (0.50, 0.70)	
**Imported drugs**				
BTD drugs	4	339	0.49 (0.32, 0.77)	.64
No BTD drugs	2	400	0.55 (0.47, 0.65)	

Abbreviations: BTD, breakthrough therapy designation; RR, response rate.

© 2024 Luo X et al. *JAMA Network Open.*

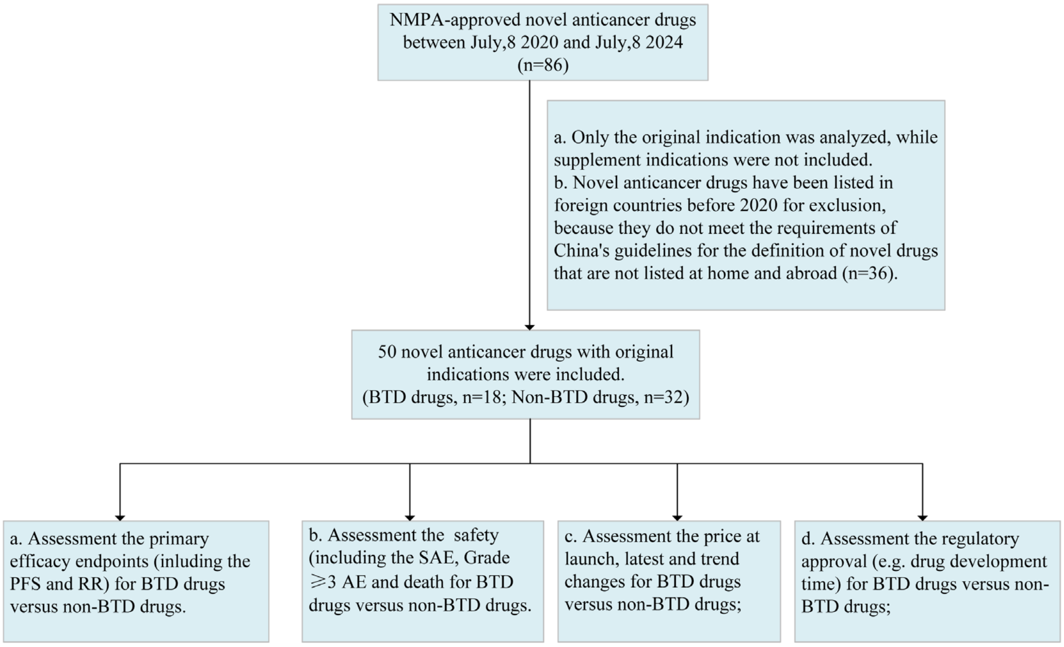

eFigure 1. Flow chart of the included cancer drugs in the analysis.

Abbreviations: NMPA, National Medical Products Administration; BTD, breakthrough therapy designation; SAE, serious adverse events; RR, response rate; PFS, progression-free survival; AE, adverse events.

Study	Events, No.	Total	RR (95%CI)	Weight, %
BTD Drugs				
Socazolimab (CCA)	14	91	0.15 (0.09 - 0.24)	2.2
Mobocertinib (NSCLC)	32	114	0.28 (0.20 - 0.37)	2.7
Cadonilimab (CCA)	32	99	0.32 (0.23 - 0.42)	2.7
Glofitamab (DLBCL)	62	154	0.40 (0.32 - 0.48)	2.9
Golidocitinib (PTCL)	39	88	0.44 (0.34 - 0.55)	2.8
Sunvozertinib (NSCLC)	59	97	0.61 (0.50 - 0.71)	3.0
Teclistamab (MM)	104	165	0.63 (0.55 - 0.70)	3.0
Glumetinib (NSCLC)	52	79	0.66 (0.54 - 0.76)	3.0
Vebreltinib (NSCLC)	39	52	0.75 (0.61 - 0.86)	3.0
Relmacabtagene (DLBCL)	45	58	0.78 (0.65 - 0.87)	3.0
Repotrectinib (NSCLC)	56	71	0.79 (0.68 - 0.88)	3.0
Linperlisib (FL)	67	84	0.80 (0.70 - 0.88)	3.1
Inaticabtagene (ALL)	31	36	0.86 (0.71 - 0.95)	3.0
Zevorcabtagene (MM)	94	102	0.92 (0.85 - 0.97)	3.1
Equecabtagene (MM)	97	101	0.96 (0.90 - 0.99)	3.1
Random effects model		1391	0.58 (0.45 - 0.74)	43.8
Heterogeneity: $I^2 = 96\%$ ($P < .001$)				
Non-BTD Drugs				
Disitamab (GC)	31	125	0.25 (0.18 - 0.33)	2.7
Tunlametinib (Melanoma)	34	95	0.36 (0.26 - 0.46)	2.8
Serplulimab (SC)	26	68	0.38 (0.27 - 0.51)	2.7
Envafolimab (SC)	44	103	0.43 (0.33 - 0.53)	2.9
Pucotenlimab (SC)	41	86	0.48 (0.37 - 0.59)	2.9
Savolitinib (NSCLC)	30	61	0.49 (0.36 - 0.62)	2.8
Tepotinib (NSCLC)	161	313	0.51 (0.46 - 0.57)	3.1
Ensartinib (NSCLC)	76	147	0.52 (0.43 - 0.60)	3.0
Pamiparib (OC)	59	101	0.58 (0.48 - 0.68)	3.0
Pralsetinib (NSCLC)	53	87	0.61 (0.50 - 0.71)	3.0
Rezivertinib (NSCLC)	146	226	0.65 (0.58 - 0.71)	3.1
Fluzoparib (OC)	79	113	0.70 (0.61 - 0.78)	3.0
Furmonertinib (NSCLC)	163	220	0.74 (0.68 - 0.80)	3.1
Olverembatinib (CML)	49	64	0.77 (0.64 - 0.86)	3.0
Unecritini (NSCLC)	89	111	0.80 (0.72 - 0.87)	3.1
Orelabrutinib (MCL)	86	106	0.81 (0.72 - 0.88)	3.1
Narlumosbart (GCT)	26	32	0.81 (0.64 - 0.93)	3.0
Penpulimab (cHL)	61	73	0.84 (0.73 - 0.91)	3.1
Zimberelimab (cHL)	79	85	0.93 (0.85 - 0.97)	3.1
Random effects model		2216	0.59 (0.51 - 0.69)	56.2
Heterogeneity: $I^2 = 94\%$ ($P < .001$)				
Random effects model		3607	0.59 (0.51 - 0.67)	100.0
Test for subgroup differences: $P = .85$				

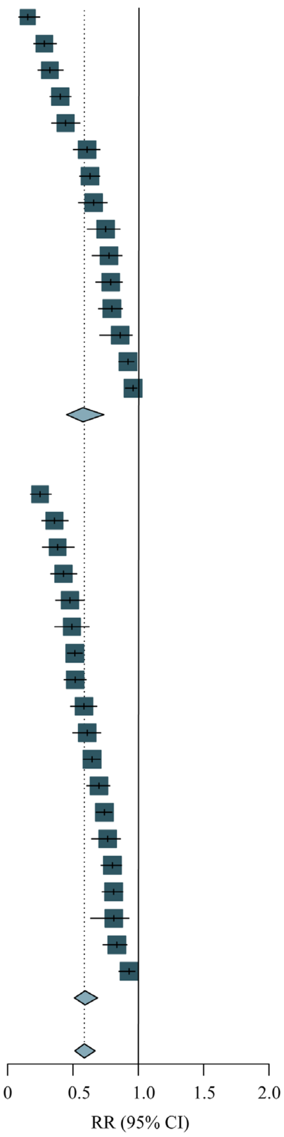

eFigure 2. Forest plot of RR as primary efficacy endpoints derived from single-arm trials for BTD versus non-BTD cancer drugs.

Confidence intervals for the RR were calculated using the Clopper-Pearson method and the variance of the pooled estimates of the proportions were stabilized using a logarithmic transformation. Vertical dashed lines indicate the results of the pooled assessment for all BTD and non-BTD drugs. NSCLC, non-small cell lung cancer; CCA, cervical cancer; DLBCL, diffuse large b-cell lymphoma; PTCL, peripheral T cell lymphomas; FL, follicular lymphoma; ALL, acute lymphoblastic leukemia; MM, multiple myeloma; GC, gastric cancer; SC, solid cancer; OC, ovarian cancer; CML, chronic myelogenous leukemia; MCL, mantle cell lymphoma; cHL, classical Hodgkin lymphoma; GCT, giant-cell tumor of bone; CI, confidence interval; RR, response rate.

© 2024 Luo X et al. *JAMA Network Open.*

Reference

1. Yang Y, Zhou J, Zhou J, et al. Efficacy, safety, and biomarker analysis of ensartinib in crizotinib-resistant, ALK-positive non-small-cell lung cancer: a multicentre, phase 2 trial. *The Lancet. Respiratory medicine.* 2020;8(1):45-53. doi: 10.1016/S2213-2600(19)30252-8.
2. Zheng J, Wang T, Yang Y, et al. Updated overall survival and circulating tumor DNA analysis of ensartinib for crizotinib-refractory ALK-positive NSCLC from a phase II study. *Cancer communications (London, England).* 2024;44(4):455-468. doi: 10.1002/cac2.12524.
3. Li N, Bu H, Liu J, et al. An Open-label, Multicenter, Single-arm, Phase II Study of Fluzoparib in Patients with Germline BRCA1/2 Mutation and Platinum-sensitive Recurrent Ovarian Cancer. *Clinical cancer research : an official journal of the American Association for Cancer Research.* 2021;27(9):2452-2458. doi: 10.1158/1078-0432.
4. Deng LJ, Zhou KS, Liu LH, et al. Orelabrutinib for the treatment of relapsed or refractory MCL: a phase 1/2, open-label, multicenter, single-arm study. *Blood advances.* 2023;7(16):4349-4357. doi: 10.1182/bloodadvances.2022009168.
5. Xu J, Shen L, Zhou Z, et al. Surufatinib in advanced extrapancreatic neuroendocrine tumours (SANET-ep): a randomised, double-blind, placebo-controlled, phase 3 study. *The Lancet. Oncology.* 2020;21(11):1500-1512. doi: 10.1016/S1470-2045(20)30496-4.
6. Gainor JF, Curigliano G, Kim DW, et al. Pralsetinib for RET fusion-positive non-small-cell lung cancer (ARROW): a multi-cohort, open-label, phase 1/2 study. *The Lancet. Oncology.* 2021;22(7):959-969. doi: 10.1016/S1470-2045(21)00247-3.
7. Wu X, Zhu J, Wang J, et al. Pamiparib Monotherapy for Patients with Germline BRCA1/2-Mutated Ovarian Cancer Previously Treated with at Least Two Lines of Chemotherapy: A Multicenter, Open-Label, Phase II Study. *Clinical cancer research : an official journal of the American Association for Cancer Research.* 2022;28(4):653-661. doi: 10.1158/1078-0432.
8. Peng Z, Liu T, Wei J, et al. Efficacy and safety of a novel anti-HER2 therapeutic antibody RC48 in patients with HER2-overexpressing, locally advanced or metastatic gastric or gastroesophageal junction cancer: a single-arm phase II study. *Cancer communications (London, England).* 2021;41(11):1173-1182. doi: 10.1002/cac2.12214.
9. Lu S, Fang J, Li X, et al. Once-daily savolitinib in Chinese patients with pulmonary sarcomatoid carcinomas and other non-small-cell lung cancers harbouring MET exon 14 skipping alterations: a multicentre, single-arm, open-label, phase 2 study. *The Lancet. Respiratory medicine.* 2021;9(10):1154-1164. doi: 10.1016/S2213-2600(21)00084-9.
10. Song Y, Zhou K, Jin C, et al. 791 A phase II study of the anti-programmed cell death-1 (PD-1) antibody penpulimab in patients with relapsed or refractory classic hodgkin lymphoma (cHL). *Journal for ImmunoTherapy of Cancer.* 2020;8(Suppl 3):A473-A474. doi: 10.1136/jitc-2020-SITC2020.0791.
11. Lin N, Zhang M, Bai H, et al. Efficacy and safety of GLS-010 (zimberelimab) in patients with relapsed or refractory classical Hodgkin lymphoma: A multicenter, single-arm, phase II study. *European journal of cancer (Oxford, England : 1990).* 2022;164:117-126. doi: 10.1016/j.ejca.2021.07.021.
12. Ying Z, Yang H, Guo Y, et al. Long-term outcomes of relmacabtagene autoleucel in Chinese patients with relapsed/refractory large B-cell lymphoma: Updated results of the RELIANCE study. *Cytotherapy.* 2023;25(5):521-529. doi: 10.1016/j.jcyt.2022.10.011.
13. Jiang Q, Huang X, Chen Z, et al. Novel BCR-ABL1 Tyrosine Kinase Inhibitor (TKI) HQP1351

(Olverembatinib) Is Efficacious and Well Tolerated in Patients with T315I-Mutated Chronic Myeloid Leukemia (CML): Results of Pivotal (Phase II) Trials. *Blood.* 2020;136:50-51. doi: 10.1182/blood-2020-142142
14. Li J, Deng Y, Zhang W, et al. Subcutaneous envafolimab monotherapy in patients with advanced defective mismatch repair/microsatellite instability high solid tumors. *Journal of hematology & oncology.* 2021;14(1):95. doi: 10.1186/s13045-021-01095-1.
15. Zhou C, Wang Z, Sun Y, et al. Sugemalimab versus placebo, in combination with platinum-based chemotherapy, as first-line treatment of metastatic non-small-cell lung cancer (GEMSTONE-302): interim and final analyses of a double-blind, randomised, phase 3 clinical trial. *The Lancet. Oncology.* 2022;23(2):220-233. doi: 10.1016/S1470-2045(21)00650-1.
16. Xu B, Zhang Q, Zhang P, et al. Dalpiciclib or placebo plus fulvestrant in hormone receptor-positive and HER2-negative advanced breast cancer: a randomized, phase 3 trial. *Nature medicine.* 2021;27(11):1904-1909. doi: 10.1038/s41591-021-01562-9.
17. Qin S, Li J, Zhong H, et al. Serplulimab, a novel anti-PD-1 antibody, in patients with microsatellite instability-high solid tumours: an open-label, single-arm, multicentre, phase II trial. *British journal of cancer.* 2022;127(12):2241-2248. doi: 10.1038/s41416-022-02043-7.
18. Gu W, Han W, Luo H, et al. Rezvilutamide versus bicalutamide in combination with androgen-deprivation therapy in patients with high-volume, metastatic, hormone-sensitive prostate cancer (CHART): a randomised, open-label, phase 3 trial. *The Lancet. Oncology.* 2022;23(10):1249-1260. doi: 10.1016/S1470-2045(22)00507-1.
19. Gao X, Xu N, Li Z, et al. Safety and antitumour activity of cadonilimab, an anti-PD-1/CTLA-4 bispecific antibody, for patients with advanced solid tumours (COMPASSION-03): a multicentre, open-label, phase 1b/2 trial. *The Lancet. Oncology.* 2023;24(10):1134-1146. doi: 10.1016/S1470-2045(23)00411-4.
20. Shi Y, Hu X, Zhang S, et al. Efficacy, safety, and genetic analysis of furmonertinib (AST2818) in patients with EGFR T790M mutated non-small-cell lung cancer: a phase 2b, multicentre, single-arm, open-label study. *The Lancet. Respiratory medicine.* 2021;9(8):829-839. doi: 10.1016/S2213-2600(20)30455-0.
21. Huang J, Song Y, Luo S, et al. Efficacy of HX008 in high microsatellite instability/mismatch repair–defificient (MSI-H/dMMR) solid tumors: Results from a multicenter phase II open-label study. *Journal of Clinical Oncology.* 2021;39(15_suppl):2572-2572. doi: 10.1200/JCO.2021.39.15_suppl.2572.
22. Qin S, Bi F, Gu S, et al. Donafenib Versus Sorafenib in First-Line Treatment of Unresectable or Metastatic Hepatocellular Carcinoma: A Randomized, Open-Label, Parallel-Controlled Phase II-III Trial. *Journal of clinical oncology : official journal of the American Society of Clinical Oncology.* 2021;39(27):3002-3011. doi: 10.1200/JCO.21.00163.
23. Wang T, Sun X, Qiu L, et al. The Oral PI3Kδ Inhibitor Linperlisib for the Treatment of Relapsed and/or Refractory Follicular Lymphoma: A Phase II, Single-Arm, Open-Label Clinical Trial. *Clinical cancer research : an official journal of the American Association for Cancer Research.* 2023;29(8):1440-1449. doi: 10.1158/1078-0432.CCR-22-2939.
24. Riely GJ, Neal JW, Camidge DR, et al. Activity and Safety of Mobocertinib (TAK-788) in Pre viously Treated Non-Small Cell Lung Cancer with EGFR Exon 20 Insertion Mutations from a Phase I/II Trial. *Cancer discovery.* 2021;11(7):1688-1699. doi: 10.1158/2159-8290.CD-23-081 2.

© 2024 Luo X et al. *JAMA Network Open.*

25. Zhou C, Ramalingam SS, Kim TM, et al. Treatment Outcomes and Safety of Mobocertinib in Platinum-Pretreated Patients With EGFR Exon 20 Insertion-Positive Metastatic Non-Small Cell Lung Cancer: A Phase 1/2 Open-label Nonrandomized Clinical Trial. *JAMA oncology.* 2021;7(12):e214761. doi: 10.1001/jamaoncol.2022.4366.
26. Wang J, Zhou C, Yao W, et al. Adebrelimab or placebo plus carboplatin and etoposide as first-line treatment for extensive-stage small-cell lung cancer (CAPSTONE-1): a multicentre, randomised, double-blind, placebo-controlled, phase 3 trial. *The Lancet. Oncology.* 2022;23(6):739-747. doi: 10.1016/S1470-2045(22)00224-8.
27. Yu Y, Zhou J, Li X, et al. Gumarontinib in patients with non-small-cell lung cancer harbouring MET exon 14 skipping mutations: a multicentre, single-arm, open-label, phase 1b/2 trial. *EClinicalMedicine.* 2023;59:101952. doi: 10.1016/j.eclinm.2023.101952.
28. Lu S, Zhou J, Jian H, et al. Befotertinib (D-0316) versus icotinib as first-line therapy for patients with EGFR-mutated locally advanced or metastatic non-small-cell lung cancer: a multicentre, open-label, randomised phase 3 study. *The Lancet. Respiratory medicine.* 2023;11(10):905-915. doi: 10.1016/S2213-2600(23)00183-2.
29. Sheng X, Ye D, Zhou A, et al. Efficacy and safety of vorolanib plus everolimus in metastatic renal cell carcinoma: A three-arm, randomised, double-blind, multicentre phase III study (CONCEPT). *European journal of cancer (Oxford, England : 1990).* 2023;178:205-215. doi: 10.1016/j.ejca.2022.10.025.
30. Shi Y, Chen J, Yang R, et al. Iruplinalkib (WX-0593) Versus Crizotinib in ALK TKI-Naive Locally Advanced or Metastatic ALK-Positive NSCLC: Interim Analysis of a Randomized, Open-Label, Phase 3 Study (INSPIRE). *Journal of thoracic oncology : official publication of the International Association for the Study of Lung Cancer.* 2024;19(6):912-927. doi: 10.1016/j.jtho.2024.01.013.
31. Li C, Wang D, Song Y, et al. CT103A, a novel fully human BCMA-targeting CAR-T cells, in patients with relapsed/refractory multiple myeloma: Updated results of phase 1b/2 study (FUMANBA-1). *Journal of Clinical Oncology.* 2023;41(16_suppl):8025-8025. doi:10.1200/JCO.2023.41.16_suppl.8025
32. Wang M, Fan Y, Sun M, et al. Sunvozertinib for patients in China with platinum-pretreated locally advanced or metastatic non-small-cell lung cancer and EGFR exon 20 insertion mutation (WU-KONG6): single-arm, open-label, multicentre, phase 2 trial. *The Lancet. Respiratory medicine.* 2024;12(3):217-224. doi: 10.1016/S2213-2600(23)00379-X.
33. Rugo HS, Im SA, Cardoso F, et al. Efficacy of Margetuximab vs Trastuzumab in Patients With Pretreated ERBB2-Positive Advanced Breast Cancer: A Phase 3 Randomized Clinical Trial. *JAMA oncology.* 2021;7(4):573-584. doi: 10.1001/jamaoncol.2020.7932.
34. Rugo HS, Im SA, Cardoso F, et al. Margetuximab Versus Trastuzumab in Patients With Previously Treated HER2-Positive Advanced Breast Cancer (SOPHIA): Final Overall Survival Results From a Randomized Phase 3 Trial. *Journal of clinical oncology : official journal of the American Society of Clinical Oncology.* 2023;41(2):198-205.doi: 10.1200/JCO.21.02937.
35. Niu X, Wei F, Tu C, et al. Efficacy and safety of JMT103 in patients with giant cell tumor of bone: A multicenter, single-arm, open-label, phase Ib/II study. *Journal of Clinical Oncology.* 2021;39(15_suppl):11526-11526. doi:10.1200/JCO.2021.39.15_suppl.11526
36. Xia Z, Leng Y, Fang B, et al. Aponermin or placebo in combination with thalidomide and dexamethasone in the treatment of relapsed or refractory multiple myeloma (CPT-MM301): a

randomised, double-blinded, placebo-controlled, phase 3 trial. *BMC cancer.* 2023;23(1):980. doi: 10.1186/s12885-023-11489-8.
37. Wang Y, Wei X, Yan D, et al. Sustained Remission and Decreased Severity of CAR T-Cell Related Adverse Events: A Pivotal Study Report of CNCT19 (inaticabtagene autoleucel) Treatment in Adult Patients with Relapsed/Refractory B-Cell Acute Lymphoblastic Leukemia (R/R B-Cell ALL) in China. *Blood.* 2022;140(Supplement 1):1598-1600.doi: 10.1182/blood-2022-165002.
38. Falchi L, Carlo-Stella C, Morschhauser F, et al. Glofitamab monotherapy in pts with relapsed/refractory (R/R) large B-cell lymphoma (LBCL): Extended follow-up and landmark analyses from a pivotal phase II study. *Journal of Clinical Oncology.* 2023;41(16_suppl):7550-7550. doi:10.1200/JCO.2023.41.16_suppl.7550.
39. Yang J-J, Zhang Y, Wu L, et al. Efficacy and safety of vebreltinib in patients with advanced NS CLC harboring MET exon 14-skipping: Results of 2.5-year follow-up in KUNPENG. *Journal of Clinical Oncology.* 2024;42(16_suppl):8557-8557. doi:10.1200/JCO.2024.42.16_suppl.855 7.
40. Mazieres J, Paik PK, Garassino MC, et al. Tepotinib Treatment in Patients With MET Exon 14 -Skipping Non-Small Cell Lung Cancer: Long-term Follow-up of the VISION Phase 2 Nonran domized Clinical Trial. *JAMA oncology.* 2023;9(9):1260-1266.doi: 10.1001/jamaoncol.2023.2 810.
41. An J, Tang J, Li BX, et al. Efficacy and Safety of the Anti-PD-L1 mAb Socazolimab for Recurrent or Metastatic Cervical Cancer: a Phase I Dose-Escalation and Expansion Study. *Clinical cancer research : an official journal of the American Association for Cancer Research.* 2022;28(23):5098-5106. doi: 10.1158/1078-0432.CCR-22-1280.
42. Chen W, Fu C, Fang B, et al. Phase II Study of Fully Human BCMA-Targeting CAR-T Cells (Zevorcabtagene Autoleucel) in Patients with Relapsed/Refractory Multiple Myeloma. *Blood.* 2022;140(Supplement 1):4564-4565. doi: https://doi.org/10.1182/blood-2022-168610.
43. Wei X, Zou Z, Zhang W, et al. A phase II study of efficacy and safety of the MEK inhibitor tunlametinib in patients with advanced NRAS-mutant melanoma. *European journal of cancer (Oxford, England : 1990).* 2024;202:114008. doi: 10.1016/j.ejca.2024.114008.
44. Lu S, Pan H, Wu L, et al. Efficacy, safety and pharmacokinetics of Unecritinib (TQ-B3101) for patients with ROS1 positive advanced non-small cell lung cancer: a Phase I/II Trial. *Signal transduction and targeted therapy.* 2023;8(1):249. doi: 10.1038/s41392-023-01454-z.
45. Xu B, Zhang Q, Hu X, et al. Entinostat, a class I selective histone deacetylase inhibitor, plus exemestane for Chinese patients with hormone receptor-positive advanced breast cancer: A multicenter, randomized, double-blind, placebo-controlled, phase 3 trial. *Acta pharmaceutica Sinica. B.* 2023;13(5):2250-2258. doi: 10.1016/j.apsb.2023.02.001.
46. Cheng Y, Chen J, Zhang W, et al. Benmelstobart, anlotinib and chemotherapy in extensive-stage small-cell lung cancer: a randomized phase 3 trial. *Nature medicine.* 2024. doi: 10.1038/s41591-024-03132-1.
47. Drilon A, Camidge DR, Lin JJ, et al. Repotrectinib in ROS1 Fusion-Positive Non-Small-Cell Lung Cancer. *The New England journal of medicine.* 2024;390(2):118-131.doi: 10.1056/NEJMoa2302299.
48. Shi Y, Wu S, Wang K, et al. Efficacy and Safety of Rezivertinib (BPI-7711) in Patients With Locally Advanced or Metastatic/Recurrent EGFR T790M-Mutated NSCLC: A Phase 2b Study.

© 2024 Luo X et al. *JAMA Network Open.*

Journal of thoracic oncology : official publication of the International Association for the Study of Lung Cancer. 2022;17(11):1306-1317. doi: 10.1016/j.jtho.2022.08.015.

49. Zhang L, Fang W, Zhao Y, et al. Ivonescimab combined with chemotherapy in patients with EGFR-mutant non-squamous non-small cell lung cancer who progressed on EGFR tyrosine-kinase inhibitor treatment (HARMONi-A): A randomized, double-blind, multi-center, phase 3 trial. *Journal of Clinical Oncology.* 2024;42(16_suppl):8508-8508. doi:10.1200/JCO.2024.42.16_suppl.8508.
50. Song Y, Malpica L, Cai Q, et al. Golidocitinib, a selective JAK1 tyrosine-kinase inhibitor, in patients with refractory or relapsed peripheral T-cell lymphoma (JACKPOT8 Part B): a single-arm, multinational, phase 2 study. *The Lancet. Oncology.* 2024;25(1):117-125. doi: 10.1016/S1470-2045(23)00589-2.
51. Zhi-Ming Li WJ, Hui Zhou, et al. Comparison of zuberitamab plus chop with rituximab plus c hop for the treatment of drug-naïve patients diagnosed with cd20-positive diffuse large b-cell l ymphoma: a phase 3 trial. *European hematology association.* 2024; 1171. June 14, 2024. Acce ssed June 14, 2024.https://library.ehaweb.org/eha/2024/eha2024-congress/419258/zhi-ming.li.comparison.of.zuberitamab.plus.chop.with.rituximab.plus.chop.for.html.
52. Shi Y, Zhang Q, Hong X, et al. Comparison of efficacy and safety of ripertamab (SCT400) versus rituximab (Mabthera(®)) in combination with CHOP in patients with previously untreated CD20-positive diffuse large B-cell lymphoma: A randomized, single-blind, phase III clinical trial. *Hematological oncology.* 2022;40(5):930-940. doi: 10.1002/hon.3054.
53. Moreau P, Garfall AL, van de Donk N, et al. Teclistamab in Relapsed or Refractory Multiple M yeloma. *The New England journal of medicine.* 2022;387(6):495-505. doi: 10.1056/NEJMoa2 203478.
54. Garfall AL, Nooka AK, Donk NWCJvd, et al. Long-term follow-up from the phase 1/2 MajesT EC-1 trial of teclistamab in patients with relapsed/refractory multiple myeloma. *Journal of Cli nical Oncology.* 2024;42(16_suppl):7540-7540. doi:10.1200/JCO.2024.42.16_suppl.7540.

© 2024 Luo X et al. *JAMA Network Open.*
