# Supplementary material for: Clinical Benefit, Price, and Regulatory Approval of Cancer Drugs Granted Breakthrough Therapy Designation in China, 2020-2024
Source: JAMA Netw Open. 2024 Oct 16;7(10):e2439080. doi: 10.1001/jamanetworkopen.2024.39080 (PMC11581593; doi:10.1001/jamanetworkopen.2024.39080)
Supplement: Supplement 2. — Data Sharing Statement [file jamanetwopen-e2439080-s002.pdf]

Data Sharing Statement

Luo. Clinical Benefit, Price, and Regulatory Approval of Cancer Drugs Granted Breakthrough Therapy Designation in China, 2020-2024. *JAMA Netw Open*. Published October 16, 2024. doi:10.1001/jamanetworkopen.2024.39080

Data
Data available: No
